# Does Air Pollution Reduce Cancer Survival? New Findings From SEER Program Cohorts

**DOI:** 10.1093/jncics/pkab004

**Published:** 2021-02-21

**Authors:** Jonathan M Samet

**Affiliations:** Office of the Dean, Department of Epidemiology, Colorado School of Public Health, Aurora, CO, USA

Air pollution causes cancer (1); does it also affect outcomes for those developing cancer? Are people with cancer at increased risk for the excess mortality associated with air pollution exposure? Innovatively using cancer patient data from the Surveillance, Epidemiology, and End Results (SEER) Program, Coleman et al. (2) address these questions in this issue of the Journal. Their findings come from 2 separate but overlapping cohorts constructed within the SEER Program database, the first composed of 5.6 million people with incident cancer and the second composed of 2.3 million 5-year cancer survivors. Long-term air pollution exposure, indexed by particulate matter less than 2.5 microns in aerodynamic diameter (PM_2.5_), was estimated for the county of residence using a model that combines regulatory modeling data with other predictors of air pollution levels. Such model estimates have now been widely used in studies of air pollution and health and have well-established accuracy (3).

Particulate matter air pollution has been causally linked to increased risk for mortality, as documented in studies from the early 1990s forward (4). Recent studies of contemporary levels of air pollution in North America and Europe continue to show that risk for premature death increases with higher exposure to PM_2.5_, particularly for cardiovascular and respiratory deaths (5). Some groups are particularly susceptible: older persons and those with cardiovascular, respiratory, and metabolic diseases. Should people with cancer and 5-year survivors be added to the populations at risk from longer term exposure to air pollution? 

For overall mortality, Coleman et al. (2) find only a very small excess risk (hazard ratio [HR]=1.01 per 10-µg/m^3^ increase in PM_2.5_, 95% confidence interval [CI] = 1.00 to 1.03), contrasting with the higher level of risk associated with PM_2.5_ observed in general population cohorts. There is a robust database from cohort studies for comparison, summarized in a recent systematic review of more than 100 such studies carried out in support of revision of the World Health Organization’s Air Quality Guidelines (5). In that review, the estimated summary relative risk (per 10 µg/m^3^ of PM_2.5_) was 1.08 (prediction interval = 1.05 to 1.11) for all-cause mortality. The contrasting findings for all-cause mortality between the cancer patient cohorts and the general population cohorts likely reflect the predominance of deaths from cancer in the full cohort (74% of deaths) and in the 5-year survivor cohort (46% of deaths). The hazard ratio for death from cancer was not increased in either cohort (HR = 0.99).

Based on the overall cohort, Coleman et al. (2) report that PM_2.5_ exposure is associated with increased risk for death from cardiopulmonary causes (HR = 1.25, 95% CI = 1.21 to 1.30), including pneumonia and influenza (HR = 1.55, 95% CI = 1.33 to 1.80). Perhaps reflecting the better health status of longer term survivors, the hazard ratios are mostly lower, but statistically significantly elevated, in the 5-year survivor cohort. For comparison, the meta-analysis provided relative risk estimates per 10-µg/m^3^ increase of PM_2.5_ of 1.11 for circulatory causes, 1.10 for respiratory causes, and 1.12 for lung cancer. The hazard ratios from the full SEER Program cohort are not uniformly higher than the comparison estimates from the meta-analysis, and the estimates for the 5-year survivors are quite close to those from the meta-analysis.

These overall analyses are complemented by explorations of heterogeneity in the association of PM_2.5_ with increased cardiopulmonary mortality across cancer types, stage and treatment, and demographics. These analyses were exploratory and compromised by smaller sample sizes within categories. Several prior reports on air pollution and cancer survival have involved people with lung cancer (6). In this report, lung cancer mortality was not increased, whereas cardiopulmonary mortality was statistically significantly increased in this subgroup of the overall cohort (2). Eckel et al. (7) examined overall survival of 352 053 lung cancer patients identified through the California Cancer Registry in relation to average air pollution exposures estimated for the residence location. The hazard ratio was statistically significantly increased for overall mortality (HR = 1.16 per 5.3-µg/m^3^ increase of PM_2.5_, 95% CI = 1.16 to 1.17), and the effect decreased for those with regional and distant stages compared with localized disease. In a 2013 report by Xu et al. (8) of respiratory cancer survival and air pollution exposure using SEER data from Los Angeles and Hawaii, PM_2.5_ was statistically significantly associated with both overall mortality and lung cancer specific mortality. Coleman et al. (2) did not stratify by SEER registry location, but such analyses could be carried out because patterns of pollution exposure vary across the SEER sites, with California residents having the highest estimated exposures.

Thus, the findings of the new study by Coleman et al. (2) represent a substantial contribution to the existing evidence on air pollution and cancer survival, which has been scant and most abundant for lung cancer. There is a single report on air pollution and survival of breast cancer patients using California SEER data (9). From a clinical perspective, the most important finding is the lack of association of overall survival of cancer patients with particulate matter air pollution, the most widely used index of ambient air pollution exposure. The SEER database used is large, and the confidence intervals around the null hazard ratio are narrow. Survival following the diagnosis of cancer has myriad determinants. The findings of Coleman et al. (2) suggest that particulate matter air pollution is not 1 of these determinants.

The authors highlight the statistically significant associations of PM_2.5_ with cardiovascular and respiratory mortality. Given the increasing number of cancer survivors, this group is yet another population at increased risk from air pollution. However, the increased risk among cancer patients and particularly among 5-year survivors is not greater than observed in general population cohorts. Nonetheless, the findings of Coleman et al. (2) confirm that air pollution continues to have adverse effects at the exposures experienced in recent decades, adding to the substantial evidence that supports the need for air quality management that reduces the health risks of air pollution.

## Funding

The author has no funding to disclose.

## Footnotes

**Role of the funder:** Not applicable.

**Disclosures:** Author has no competing interests to disclose.

## Data Availability

Not applicable.
